# Children in child protective services custody experience higher risk of intramuscular medication and physical restraints in a pediatric emergency department

**DOI:** 10.1371/journal.pone.0355397

**Published:** 2026-08-12

**Authors:** Isaac V. Faustino, Max J. Rolison, Ambrose H. Wong, Pamela Hoffman, Veronika Shabanova, Gunjan Tiyyagura

**Affiliations:** 1 Department of Emergency Medicine, Yale School of Medicine, New Haven, Connecticut, United States of America; 2 Child Study Center, Yale School of Medicine, New Haven, Connecticut, United States of America; 3 Department of Psychiatry and Behavioral Sciences, Boston Children’s Hospital, Boston, Massachusetts, United States of America; 4 Ronald O. Perlman Department of Emergency Medicine, NYU Grossman School of Medicine, New York, New York, United States of America; 5 Department of Biostatistics, Yale School of Public Health, New Haven, Connecticut, United States of America; 6 Department of Pediatrics, Yale School of Medicine, New Haven, Connecticut, United States of America; NYU Grossman School of Medicine: New York University School of Medicine, UNITED STATES OF AMERICA

## Abstract

**Objective:**

To assess the association between child protective services (CPS) involvement and the risk and timing of intramuscular (IM) medication or physical restraint use in pediatric patients presenting to the emergency department (ED) with behavioral health concerns.

**Methods:**

An electronic health record dataset spanning January 2021 to October 2023 was retrospectively analyzed. Individual cumulative incidence plots and Cox regression models were created to model the risk and risk rate of receiving either IM medication or being physically restrained by different levels of CPS involvement. Cox regression models were adjusted for age, legal sex, and race and ethnicity.

**Results:**

We analyzed 6257 behavioral health encounters. Children in CPS custody had the highest cumulative incidence of receiving IM medication or being physically restrained compared to children with a new CPS report filed or children without any CPS involvement after 72 hours (IM medication: 17%, 6%, 5% respectively, p < 0.001, physical restraints: 8%, 4%, 3% respectively, p < 0.001). Compared to children without CPS involvement, children in CPS custody had a significantly higher risk rate of receiving IM medications (adjusted hazard ratio (aHR) 1.79, 95% Confidence Interval (CI) 1.01–3.18, p = 0.046), and physical restraints (aHR 2.71, 95% CI 1.35–5.43 p = 0.005).

**Conclusion:**

Children in CPS custody had a higher risk of receiving IM medication and being physically restrained during behavioral health encounters in a pediatric ED. Further work should focus on understanding the causal mechanisms and designing interventions that maximize optimal health outcomes for vulnerable children experiencing behavioral health emergencies.

## Introduction

Behavioral health visits to pediatric emergency departments (ED) have risen substantially, increasing from 4.8 million to 7.5 million between 2011 and 2020 [1]. During these encounters, patients may become acutely agitated, placing themselves or others at risk of harm [2]. While verbal de-escalation and behavioral techniques are recommended as first line interventions, more restrictive interventions, including intramuscular (IM) medication or physical restraints, may be used to prevent harm [3]. In the United States, 6–10% of youth seeking emergency behavioral health care are subjected to physical restraint use [4,5]. While IM medication and physical restraints are used to maintain safety, they carry meaningful risks such as physical injury, psychological distress, medication-related adverse effects, and in rare cases, death [3].

In a previous study, we found that encounters with any child protective services (CPS) involvement had 1.91 times higher odds of physical restraint use [6]. These findings raise concern that children with CPS involvement may receive differential care during behavioral health emergencies. However, important gaps remain. Prior work treated CPS involvement as a single category, without distinguishing between children with a new CPS report filed and those in CPS custody, despite meaningful differences in clinical and social circumstances between these groups. A new CPS report filed during the ED visit reflects an acute concern identified at presentation, whereas patients already in CPS custody have pre-existing child welfare involvement and likely greater cumulative adversity exposure. These contexts differ in baseline risk, supervision structure, and disposition constraints, which may independently influence physical restraint use.

Furthermore, existing studies have focused primarily on whether IM medication or physical restraint occurs, rather than when it occurs during an encounter. Time-to-event analysis may provide important insight into behavioral escalation processes in the ED. Understanding timing is particularly important for children in CPS custody, who often experience prolonged ED boarding while awaiting placement. If risk increases with length of stay, this may suggest opportunities for earlier disposition coordination or the proactive implementation of trauma-informed therapeutic services to mitigate escalation.

This study builds on prior evidence by examining how the level of CPS involvement, specifically how having a new CPS report filed versus being in CPS custody, is associated with both the timing and use of IM medication and physical restraints during pediatric behavioral health emergency care. Examining these associations is an essential first step prior to focusing on causal mechanisms for future work in modifiable factors and developing equitable, systems-level interventions to reduce harm among children experiencing behavioral health crises.

## Methods

### Study design and setting

We conducted a retrospective cohort study using electronic health records of pediatric ED encounters collected from a single tertiary care children’s hospital which has an annual volume of 38,979 visits with roughly 1,900 (4.87%) annual visits involving children younger than 16 years old presenting with behavioral health concerns. The Yale University Human Investigation Committee considered this study exempt from IRB review. Data was accessed when this project started in November 2024. Analyses were done using a truncated dataset that excluded identifiable patient information.

### Study population

All pediatric ED encounters with behavioral chief complaints between January 1, 2021 and October 31, 2023 were included in the analysis. Behavioral health presentations were defined as the presence of mental health, intoxication, and/or poisoning-related chief concerns or associated with psychiatric disorders and intoxications (Table 1). Patients younger than 5 years old were excluded, given developmental differences in presentation and the rarity of IM medication or physical restraint use in this age group.

**Table 1:** Behavioral health presentations based on chief concern.

Chief Concern
• Psychiatric Evaluation • Aggressive Behavior • Suicidal • Agitation • Behavior Problem • Combative • Homicidal • Hallucinations • Suicide Attempt • Psychotic Symptoms • Anxiety • Self-Mutilation • Depression • Manic Behavior • Panic Attack • Mental Health Problem • Tics • Paranoid • Psychosis • Runaway • Poisoning • Alcohol Intoxication • Overdose- Intentional • Drug Problem, **•** Overdose-Accidental **•** Drug/Alcohol Assessment **•** Addiction Problem **•** Alcohol Use

### Primary exposure variable

Encounters were categorized into one of three mutually exclusive groups based on CPS level of involvement: (1) no CPS involvement, (2) a new CPS report filed during the ED visit only, or (3) a patient in CPS custody. If a patient was in CPS custody and had a new report filed, the encounter was categorized in the CPS custody group.

We elected to maintain this categorization specifically because being in CPS custody represents the patient’s existing legal status and care environment at the time of ED encounter. Additionally, children in CPS Custody with a new report filed remain in CPS custody and continue to share key characteristics relevant to our study question, including the same guardian/legal decision-maker structure, placement-related challenges, fractured behavioral healthcare, and child welfare oversight.

### Outcome measures

The co-primary outcomes during the first 72-hours of ED admission were: (1) cumulative incidence and risk rate of receiving IM medication, and (2) cumulative incidence and risk rate of receiving physical restraint.

IM medication administration and physical restraint use were identified from the electronic health record (EHR). Manual chart review was conducted by a study team member (IVF) for encounters that included a physically restrained patient by reviewing an encounter’s chart to accurately capture timing of physical restraint use and to verify whether a physical restraint was placed in the ER or by EMS [7]. When capturing these details was unclear, a second reviewer (MJR) independently reviewed the same documentation and discrepancies were resolved by consensus.

For descriptive purposes, time to first physical restraint was additionally categorized as: (1) physical restraint present on arrival, (2) applied within 30 minutes of ED arrival, or (3) applied more than 30 minutes after arrival. We selected the 30-minute cutoff because we believed physical restraints used in the first 30 minutes were more likely driven by pre-hospital circumstances, transport-related factors or the transition into the ED environment, leaving limited opportunities for ED-based interventions to affect outcomes. After 30 minutes, we considered physical restraints more likely to be influenced by factors related to the ED environment. Additionally, we examined the distribution of time from arrival to restraint and found that there was a natural clustering of early restraint events before 30 minutes. Although somewhat arbitrary, we selected 30 minutes as a clinically reasonable threshold that balanced the previous considerations.

### Covariates

Models were adjusted for patient-level demographics selected a priori based on literature and clinical relevance: age, legal sex, and race and ethnicity [6,8–11].

### Analysis

We converted the cumulative survival probability of not receiving IM medication or being physically restrained, S(t), to the cumulative distribution function F(t)=1-S(t) to directly model the cumulative incidence (risk by a certain time point) using the number and timing of events where IM medication was given or physical restraints were used, separately for each outcome.

In unadjusted analyses, cumulative incidence plots were constructed for each CPS group. These plots model the number of events where IM medication was given or physical restraints were used across a 72-hour time period. Plots were first tested for significant differences in at least one pair of CPS groups using a Peto-Peto weighted log rank test [12]. This was followed by a Peto-Peto weighted pairwise comparison test to determine which specific pair of CPS group curves was significantly different from each other.

Separately for each outcome of interest, Cox proportional hazards regression models with patient-specific frailty terms to account for repeat ED encounters were built to estimate adjusted hazard ratios (aHR), 95% Confidence Interval (CI) for IM medication or physical restraint by CPS group, adjusting for *a priori* chosen covariates [13]. Encounters with patients without CPS involvement were used as the reference group. The proportional hazards assumption was found to not be violated after evaluating it using Schoenfeld residuals. Since overall missingness across the outcomes of interest and covariates was < 0.3%, we categorized encounters with missing race and ethnicity information as “Other” and proceeded to analyze all encounters. We did not adjust our analyses for multiple comparisons because (1) our co-primary outcomes tested independent and prespecified hypotheses of interest, rather than repeated tests of a single effect and (2) while we chose the no CPS involvement category as our reference group, the two between-group comparisons (CPS custody versus no CPS involvement and a new CPS report filed only versus no CPS involvement) were limited by the census of the available data from our institution, thus we have chosen to focus on using 95% CIs to support interpretation [14]. Hypothesis testing was performed at a 2-sided level of significance of 0.05. Analyses were conducted using R version 4.4.2.

## Results

Among 6257 behavioral health encounters consisting of 3837 unique patients, 2628 encounters (42.0%) included patients identifying as male at birth. The average patient age among all included encounters was 12.9 years (SD: 2.8). IM medication was given in 200 (3.2%) encounters and physical restraints were used in 109 encounters (1.7%). Among all encounters, 111 (1.8%) received only IM medication, 20 (0.3%) were physically restrained, 89 (1.4%) received both IM medication and were restrained, and 6037 (96.5%) needed neither.

There were 5347 (85.5%) encounters where the patient did not have CPS involvement, 474 (7.6%) encounters where a new CPS report was filed, and 436 (6.9%) encounters where the patient was in CPS custody (Table 2). Of the 436 encounters involving patients in CPS Custody, 185 (42.4%) also had a new CPS report filed during the encounter. Mean lengths of stay for encounters with no CPS involvement, encounters with a new CPS report filed, and encounters with patients in CPS custody were 19.8 hours (SD: 27.4), 30.0 hours (SD: 37.0), and 33.6 hours (SD: 70.3) respectively (Table 2).

**Table 2:** Demographic and visit characteristics of pediatric ED visits based on level of CPS involvement.

N = 6257	No CPS Involvement n = 5347	CPS Report Only n = 474	CPS Custody n = 436
**Mean age, years (SD)**	13.0 (2.8)	12.6 (2.3)	11.6 (2.6)
**Legal Sex, n (%)**			
*Female*	3,112 (58.2%)	290 (61.2%)	227 (52.1%)
*Male*	2,235 (41.8%)	184 (38.8%)	209 (47.9%)
**Race and Ethnicity,** **n (%)**			
*White non-Hispanic*	2,386 (44.6%)	159 (33.5%)	109 (25.0%)
*Black non-Hispanic*	1,153 (21.6%)	124 (26.2%)	183 (42.0%)
*Hispanic*	1,524 (28.5%)	169 (35.7%)	136 (31.2%)
*Other*	284 (5.3%)	22 (4.6%)	8 (1.8%)
**Primary Language, n (%)**			
*English Speaking*	4,998 (93.5%)	444 (93.7%)	432 (99.1%)
*Non-English Speaking*	349 (6.5%)	30 (6.3%)	4 (0.9%)
**Insurance, n (%)**			
*Private Insurance*	2,330 (43.6%)	97 (20.5%)	23 (5.3%)
*Public Insurance*	3,017 (56.4%)	377 (79.5%)	413 (94.7%)
**Arrival Method,** **n (%)**			
*Car/Walk-in*	3,010 (56.3%)	160 (33.8%)	91 (20.9%)
*Police or EMS*	2,077 (38.8%)	281 (59.3%)	305 (70.0%)
*Not Specified*	260 (4.9%)	33 (7.0%)	40 (9.2%)
**Mean ED Length of Stay, hours (SD)**	19.8 (27.4)	30.0 (37.0)	33.6 (70.3)
IM Medication Used*			
*No*	5,198 (97.2%)	455 (96.0%)	404 (92.7%)
*Yes*	149 (2.8%)	19 (4.0%)	32 (7.3%)
**Physical Restraints** Used*			
*No*	5,271 (98.6%)	462 (97.5%)	415 (95.2%)
*Yes*	76 (1.4%)	12 (2.5%)	21 (4.8%)
**Physical Restraint Timeframe**			
*Arrival*	14 (0.3%)	3 (0.6%)	4 (0.9%)
*First 30 mins*	13 (0.2%)	1 (0.2%)	2 (0.5%)
*Later than 30 mins*	49 (0.9%)	8 (1.7%)	15 (3.4%)
*Not applicable*	5,271 (98.6%)	462 (97.5%)	415 (95.2%)

CPS = child protective services, EMS = emergency medical services, IM = intramuscular.

The “Other” race and ethnicity categories includes those who identified as Asian American, American Indian or Native American, Native Hawaiian or Other Pacific Islanders, Guamanian or Chamorro, and those who preferred not to share, do not know, do not see their race listed, or had missing race demographic information.

*Raw percentage.

There were 1049 unique patients that had at least 2 behavioral health encounters. We found that 18 patients (23.4% of 77 unique patients in CPS custody), 13 patients (12.4% of 105 unique patients with only a new CPS report) and 45 patients (5.2% of 867 unique patients with no CPS involvement) had a prior history of IM medication or physical restraint. A chi-square test revealed that CPS grouping and prior history were associated (p < 0.001).

**Fig 1:**
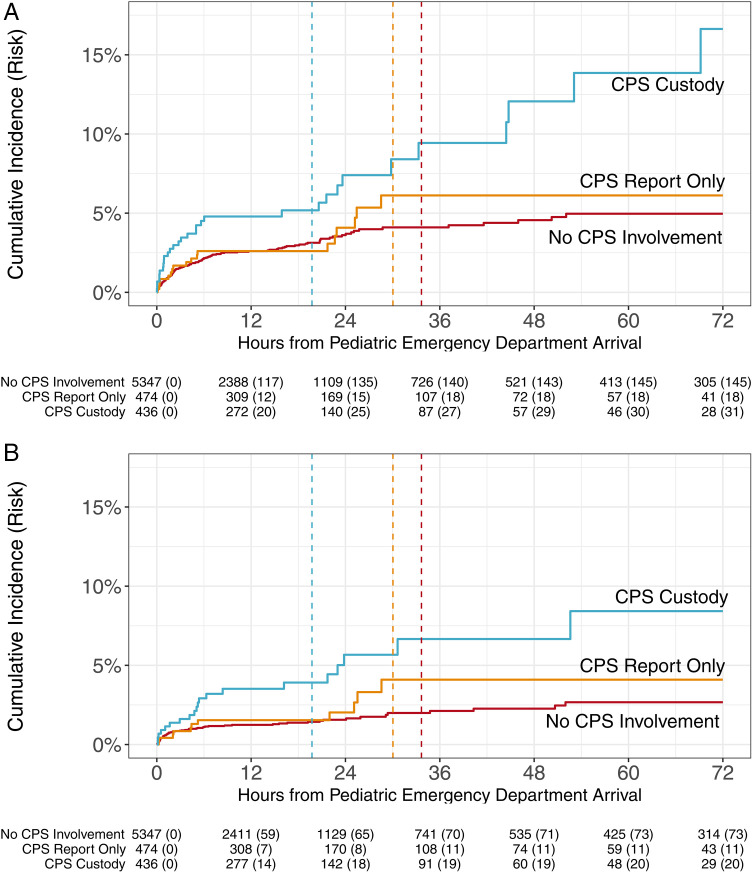
Cumulative incidence of (A) IM medication and (B) physical restraint by CPS group up to 72 hours after arrival. Children in CPS custody had the highest cumulative incidence of IM medication and physical restraint. Mean length of stay for each group are indicated by the dashed vertical line of the same color. Numbers in the table represent patients at risk at each time point (cumulative number of patients (A) IM medicated or (B) physically restrained).

Encounters with patients in CPS custody had the highest cumulative incidence of IM medication being given across a 72-hour time period (Fig 1A). The cumulative incidences at 24 hours were 135 (4%) encounters with patients without CPS involvement, 15 (4%) encounters with patients with a new CPS report filed, and 25 (7%) encounters with patients in CPS custody were given IM medication (p < 0.001). By 48 hours, the cumulative incidences increased to 143 (5%), 18 (6%), and 29 (12%) respectively (p < 0.001). Finally at 72 hours, the cumulative incidences increased to 145 (5%), 18 (6%), and 31 (17%) respectively (p < 0.001). A Peto-Peto weighted pairwise comparison test demonstrated a significant difference between the cumulative incidence curves involving encounters with patients with no CPS involvement and encounters with patients in CPS custody (p < 0.001) as well as between encounters with patients with a new CPS report filed and encounters with patients with CPS custody (p = 0.031). Among encounters where IM medication was given, the mean time to IM medication was 6.84 hours (SD: 10.13) for encounters with patients without CPS involvement, 9.63 hours (SD: 10.94) for encounters with a new CPS report filed, and 13.56 hours (SD: 18.50) for encounters with patients in CPS custody.

Encounters with patients in CPS custody also had the highest cumulative incidence of physical restraint use over a 72-hour time period (Fig 1B). The cumulative incidences at 24 hours were 65 (2%) encounters with patients without CPS involvement, 8 (2%) encounters with patients with a new CPS report filed, and 18 (6%) encounters with patients in CPS custody were restrained (p < 0.001). By 48 hours, the cumulative incidences increased to 71 (2%), 11 (4%), and 19 (7%) respectively (p < 0.001). Finally at 72 hours, the cumulative incidences increased to 73 (3%), 11 (4%), and 20 (8%) (p < 0.001). A Peto-Peto weighted pairwise comparison test revealed a significant difference only between the cumulative incidence curves involving encounters with patients without CPS involvement and encounters with patients in CPS custody (p < 0.001). Among encounters where physical restraints were used, the mean time to being restrained was 6.92 hours (SD: 11.80) for encounters with patients without CPS involvement, 10.90 hours (SD: 11.62) for encounters with a new CPS report filed, and 10.69 hours (SD: 13.56) for encounters with patients in CPS custody.

From an adjusted Cox proportional hazards model where IM medication was given (Table 3), encounters with a new CPS report filed did not show an increased risk rate (aHR 1.09, 95% CI, 0.65–1.85, p = 0.7) relative to encounters without CPS involvement. Encounters with patients in CPS custody demonstrated an increased risk rate (aHR 1.79, 95% CI, 1.01–3.18, p = 0.046) relative to encounters without CPS involvement. From an adjusted Cox proportional hazards model where physical restraints were used (Table 3), encounters with a new CPS report filed did not show an increased risk rate (aHR 1.31, 95% CI, 0.67–2.57, p = 0.4) relative to encounters without CPS involvement. Encounters with patients in CPS custody demonstrated an increased risk rate (aHR 2.71, 95% CI, 1.35–5.43, p = 0.005) relative to encounters without CPS involvement.

**Table 3:** Hazard ratios from separate unadjusted and adjusted Cox regression models for IM medication and physical restraint.

	CPS Group	Unadjusted Model HR (95% CI)	Adjusted Model aHR (95% CI)
**IM Medication**	*No CPS Involvement*	Ref.	Ref.
	*CPS Report Only*	1.11 (0.66, 1.89)	1.09 (0.65, 1.85)
	*CPS Custody*	1.96 (1.12, 3.44)	1.79 (1.01, 3.18)
**Physical Restraint**	*No CPS Involvement*	Ref.	Ref.
	*CPS Report Only*	1.35 (0.69, 2.66)	1.31 (0.67, 2.57)
	*CPS Custody*	2.89 (1.46, 5.71)	2.71 (1.35, 5.43)

CPS = child protective services, HR = hazard ratio, aHR = adjusted hazard ratio, IM = intramuscular. Models were adjusted for age, sex, and race and ethnicity.

### Limitations

These findings should be understood in the context of several limitations. Given the nature of the EHR, we recognize that race and ethnicity, CPS classification, first IM medication, and first physical restraint order time may be captured inaccurately due to incorrect EHR data variable collection and pertinent information not captured in an encounter’s chart. Additionally, due to this study’s retrospective nature, we were unable to reliably capture other potentially relevant clinical information such as the severity of behavioral crisis, prior trauma experienced, staffing variability, and whether a supportive caregiver was present. These factors may be confounders and could affect the risk of receiving either IM medication or physical restraint. Finally, these findings were from a single study site that only included patients with behavioral health concerns.

## Discussion

We report two main findings in this study. Among children seeking care in a pediatric ED for behavioral health concerns, we found that children in CPS custody had a higher cumulative incidence of receiving IM medication or being physically restrained over a 72-hour time period starting upon arrival to the pediatric ED compared to children with a new CPS report filed or no CPS involvement. Similarly, in adjusted analysis, children in CPS custody had significantly higher hazards of receiving IM medication or being physically restrained, even after accounting for demographic factors.

By distinguishing children in CPS custody from those with a new CPS report filed, our study demonstrates that not all CPS involvement confers equal risk; rather, children already in custody appear uniquely vulnerable to more restrictive interventions during behavioral health crises. The higher rates of IM medication and physical restraint use at different time points throughout the ED encounter suggest that these children may be at risk for more rapid escalation in the ED environment. Identifying this subgroup as disproportionately affected highlights an urgent need for targeted, trauma-informed interventions, proactive de-escalation strategies, and systems-level approaches, such as expedited disposition planning or early behavioral health support to reduce reliance on restrictive measures and mitigate harm.

Children in CPS custody may experience increased frequency of IM medication or physical restraints use for multiple systemic or structural reasons. First, children in CPS custody often have extensive trauma histories, fractured care, and disrupted placements, factors that may contribute to escalation or influence clinical decision-making or treatment. Children in CPS custody are more likely to have accumulated adverse childhood experiences which frequently lead to various internalizing and externalizing behaviors [6,15,16]. Consistent with this explanation, children with CPS involvement in our study were significantly more likely to have a prior history of IM medication administration or physical restraint use than youth without CPS involvement. Second, children in CPS custody frequently experience fractured care or lack access to timely care, such as missed medication doses, potentially leading to increased episodes of agitation requiring ED care [16–20]. Third, it has been previously reported that Black youth have substantially higher rates of CPS contact than White children, which may ultimately lead Black youth to being placed in the CPS custody [21,22]. Additionally, in our study Black youth independently experience higher risk of restraint use compared to other races, consistent with the broader literature [6,23,24]. Implicit biases resulting from the intersection of being in CPS custody and race may also contribute to the challenges experienced by Black youth in CPS custody. Finally, placement instability with frequent moves between foster homes can cause behavioral dysregulation, manifesting as aggression which can then lead to increased physical restraint use [16,19,20]. Unfortunately, these episodes may contribute to further disruptions in placement, as families may be unable to care for children with significant behavioral health needs and decline to have the child return, resulting in subsequent placement in a new home, thereby perpetuating a cycle of instability [16]. Significant behavioral dysregulation episodes may cause a child to go to the ED, which may lead these episodes to become further amplified [6,10,19,25].

Once in the ED, children in CPS custody may experience increased IM medication and physical restraint use for various reasons. EDs are frequently crowded and under-resourced with a focus on patients presenting with acute medical issues [1,26]. ED staff also may have insufficient training in trauma-informed behavioral health care which is compounded by a shortage of providers with behavioral health expertise [15,27–29]. Limited access to quiet, confidential spaces and resources to create a therapeutic environment can further traumatize children in CPS custody, who have already experienced significant trauma [26,30]. Additionally, long waits for the evaluation and prolonged boarding times while awaiting placement for higher levels of care, which our study found to be worse for children in CPS custody, may be a further setup for behavioral dysregulation and increased physical restraint use [26,31,32]. Furthermore, children in CPS custody often lack a trusted advocate who can identify triggers, suggest mitigating strategies, and offer support [33,34]. For children in CPS custody, delays in obtaining authorization for non-emergent medications, sometimes up to 48 hours in our experience, may also limit early and preventative therapeutic intervention, increasing the likelihood of escalation to IM medications or crisis situations [32–34]. Next steps include assessing the mechanisms linking CPS custody status to increased physical restraint use, and to particularly quantify the independent contributions of systemic factors (ED crowding, resource availability) versus patient-level factors (trauma burden, behavioral acuity).

Decreasing physical restraint use and length of stay among children in CPS custody is multifactorial and includes efforts to improve care processes both within the ED and in the community. As behavioral health presentations are projected to increase, expanding a workforce experienced in behavioral health care and training staff in trauma-informed approaches may improve de-escalation practices and reduce the use of physical restraints [1]. Pediatric EDs can also improve care by adopting physical and logistical changes that support low-stimulation environments. Overcrowding and the absence of quiet, engaging activities during long waits may contribute to behavioral dysregulation, whereas providing dedicated spaces and calming activities may reduce anxiety [26,27,32]. Additionally, children in CPS custody often lack supportive caregivers to advocate for them during emergency care. The presence of a consistent, supportive adult may reduce anxiety, decrease the need for physical restraints, and provide companionship during prolonged or uncertain hospital stays. Future studies could examine how caregiver presence influences patient outcomes.

Community-based alternatives may help prevent behavioral health-related ED visits and maintain CPS-involved children in the least restrictive settings. Mobile crisis services, which connect patients to community-based supports, have demonstrated cost-effectiveness and positive clinical outcomes in adults and have been associated with reduced subsequent behavioral health ED visits in pediatric populations [35]. Schools may also play a role in early identification and referral to appropriate behavioral health resources [33]. Telehealth offers an additional alternative by improving access to care, offering continuity of care with current providers, and avoiding ED-specific challenges such as travel, prolonged boarding, and risk of physical restraint [29,30,33,36]. Together, these approaches may reduce unnecessary pediatric ED utilization and should be explored in future studies. However, acute agitation and safety concerns may still necessitate ED care.

## Conclusion

We provide evidence that children in CPS custody have a higher risk of receiving IM medication and being restrained within 72 hours of arriving to the pediatric ED compared to children with a new CPS report filed or no CPS involvement. Next steps include exploring the causes of these differences in behavioral health care and evaluating both ED and community-based interventions to improve care among CPS-involved children.

## Supporting information

S1 Data Dataset for IM medication cumulative incidence curves. (CSV)

S2 Data Dataset for physical restraint cumulative incidence curves. (CSV)
