# Supplementary material for: Children in child protective services custody experience higher risk of intramuscular medication and physical restraints in a pediatric emergency department
Source: PLoS One. 2026 Aug 12;21(8):e0355397. doi: 10.1371/journal.pone.0355397 (PMC13465842; doi:10.1371/journal.pone.0355397)
Supplement: Supplementary file 1 [file pone.0355397.s004.docx]

The comments provided by the reviewers have greatly strengthened our manuscript and we thank you for the opportunity to resubmit our work for consideration in PLOS One.

| Reviewer Comments | Response | Changes to the Manuscript |
| --- | --- | --- |
| **Reviewer 1** | | |
| The authors conducted a single center retrospective cohort study using electronic health records from pediatric ED encounters, showing that children in CPS custody had higher risk of receiving IM medication and physical restraint during BH encounters.    I appreciate the focus on the important topic of children under CPS care and the unique strength of the time-to-event analysis. | Thank you for your summary and appraisal of our study. | No changes. |
| My primary concern with the findings is confounding by acuity. In other words, are children in CPS or children that receive a CPS consult more acutely or chronically ill and therefore restrictive interventions were clinically warranted/justified in such cases?  I understand the authors’ position that this paper is an initial step and not, in theory, pointing to causality, but the discussion focuses on the hypothesis that CPS care places children at risk for restrictive interventions (which might be true) but spends less time on the idea that children placed within CPS care are already more acutely and chronically ill in the first place.  It would be interesting to see if those who have a CPS consult or are in CPS custody have a history of restraint or IM sedation in prior visits. If not, this would strengthen the authors contention. | We appreciate the reviewer’s thoughtful comment. We agree that children in CPS custody or children with new CPS referrals may have more frequent and severe BH dysregulation requiring additional restrictive interventions.  Per the reviewer’s recommendation, we examined patients who had at least 2 encounters in our health system and analyzed whether the CPS grouping of a patient’s most recent encounter was associated with having a prior IM medication or physical restraint event. We found that 18 patients (23.4% of 77 unique patients in CPS Custody), 13 patients (12.4% of 105 unique patients with only a new CPS report), and 45 patients (5.2% of 867 unique patients with no CPS involvement) had a prior history of IM medication or physical restraint. A chi-square test revealed that CPS grouping and prior history of IM medication or physical restraint were associated (p<0.001).  This supplemental analysis suggests that youth in CPS custody and those with new CPS referrals were significantly more likely to have a prior history of IM medication administration or physical restraint than youth without CPS Involvement. This finding supports the possibility that these populations enter emergency care settings with greater baseline behavioral health complexity and vulnerability. However, such differences may themselves reflect the cumulative effects of adverse childhood experiences, maltreatment exposure, placement instability, and fragmented access to behavioral health services that are disproportionately experienced by CPS-involved youth. Therefore, we believe that greater clinical acuity and structural vulnerability should be viewed as complementary rather than competing explanations for the observed associations. | We added this supplemental analysis about patients with prior history of IM medication or physical restraint events and their CPS grouping in the results section on **lines 173 - 177.**  We have also added the following in the discussion **lines 269-272**:  Consistent with this explanation, children with CPS involvement in our study were significantly more likely to have a prior history of IM medication administration or physical restraint use than youth without CPS Involvement. |
| 1. In table 1 can the authors explain the inclusion of “hyperventilating?” Seems like there would be a broad overlap with acute medical presentations. | Hyperventilating was used as a chief complaint to capture instances of acute anxiety. We removed 25 encounters from the analysis dataset that listed “hyperventilating” as the primary chief complaint but did not have an accompanying acute anxiety chief complaint. | We removed “hyperventilating” from Table 1.  Although a minor change, we updated Table 2, analyses and results throughout the manuscript to reflect the removal of these encounters. |
| 2. Can the authors give one line as to why you chose to group CPS custody + new report into the custody group vs. creating a fourth group? At minimum, could authors report how many of these there were? | We appreciate the reviewer’s suggestion. We categorized encounters involving children already in CPS custody and a new CPS report within the CPS custody group because CPS custody represents the child’s existing legal status and care environment at the time of the ED encounter. Although a new report may be filed, these children remain in CPS custody and continue to share key characteristics relevant to our study question, including the same guardian/legal decision-maker structure, placement-related challenges, fractured behavioral healthcare, and child welfare oversight. Therefore, we considered CPS custody to be the dominant exposure category.  Of the 436 encounters involving patients in CPS custody, 185 (42.4%) also had a new CPS report filed during the encounter. | We added information to clarify our rationale for classifying these encounters within the CPS custody group in the methods section on **lines 101 – 106**.  We added the number of encounters with patients in CPS custody receiving a new CPS referral in the results section on **lines 168 – 169.** |
| 3. I appreciate the authors using manual review of charts to determine timing of physical restraint use. Is there precedent for this? Curious to know how accurate/validated this methodology is? | There is precedence for using manual chart review to identify and characterize restraint events, although this approach is not uniformly described or validated across studies. In a recent systematic review of restrictive care practices, a subset of studies used manual review of medical records to extract restraint-related data, including timing and duration of restraint episodes (Belayneh et al. 2024). However, the review also highlighted substantial variability in reporting and a lack of formal validation of measurement approaches across studies.  In our study, we used manual chart review to determine the timing of physical restraint use because structured elements alone did not consistently capture this information. For each encounter, in which physical restraint use was documented, a study team member (IVF) reviewed clinician and nursing documentation to verify whether restraints were used and to identify the timing of the restraint event relative to the emergency department encounter. In cases where the timing was unclear or not explicitly documented, a second reviewer (MJR) independently reviewed the same documentation and discrepancies were resolved by consensus. | We have added this clarification regarding our manual chart review process briefly in the methods section on **lines 112 – 117**.  We also acknowledged the limitations of this in the limitations section on **lines 239 – 240**. |
| **Reviewer 2** | | |
| Overall, this is a well-written, clinically crucial, and methodologically sound retrospective cohort study. It addresses a major gap in pediatric emergency medicine and child welfare literature by breaking down "CPS involvement" into nuanced categories (custody vs. new report) and utilizing time-to-event analysis.  The review below is organized into major and minor revisions to help the authors improve the manuscript before publication. | Thank you for your summary and appraisal of our study. | No changes. |
| Reviewer Scientific Evaluation Report Recommendation: Major Revision  General Assessment The manuscript tackles an incredibly vital topic—the intersection of child welfare status and restrictive clinical interventions (physical restraints and intramuscular chemical restraints) in pediatric emergency departments. The use of Cox proportional hazards models with patient-specific frailty terms to account for repeat visits is a robust and appropriate statistical choice.  However, there is a striking demographic signal in Table 2 regarding Race and Ethnicity that requires immediate attention. The authors adjust for race/ethnicity in their regression models as a confounder, but they completely omit any discussion of how racial disparities overlap with CPS custody status and restraint use in their Discussion section. This must be addressed to ensure the study provides a complete, systems-level perspective. | We appreciate your assessment of our study and have answered each of your major and minor revision comments below. | Please see below. |
| Major Revisions | | |
| 1. Address Intersectionality and Racial Disparities in the Discussion  In Table 2, the demographic breakdown of the cohorts reveals a severe racial imbalance across the levels of CPS involvement: White non-Hispanic: Dropping from 44.5% (No CPS) to 25.0% (CPS Custody). Black non-Hispanic: Surging from 21.6% (No CPS) to 42.0% (CPS Custody).  We know from existing literature (including the authors' cited reference, Nash et al., 2021) that Black youth face a disproportionately higher risk of being physically restrained in emergency settings. By adjusting for race in the Cox model, the authors show that CPS custody carries an independent risk (aHR = 2.72 for restraints), but they fail to discuss how systemic racism in child welfare pipelines concentrates highly vulnerable Black youth into this highest-risk "CPS Custody" cohort.  Action Required: Add a dedicated subsection or paragraph in the Discussion exploring this intersection. The paper cannot fully evaluate "systems-level interventions" without acknowledging how structural racism impacts both child welfare entry and clinical management in behavioral crises. | We appreciate the reviewer’s suggestion to incorporate information exploring how racial inequities leads to Black youth disproportionately being concentrated within CPS custody.  We revisited the literature to write additional information about this phenomenon and how it connects to our study’s findings. | We have included additional information describing how Black youth have disproportionately higher rates of CPS involvement and how this is associated with higher rates of IM medication and physical restraint use within the pediatric ED environment in the discussion section **on lines 274 - 280.** |
| 2. Discrepancy Between Abstract and Results Text  There is a confusing numerical discrepancy between the cumulative incidence rates reported in the Abstract versus the Results section text:  Abstract: Reports 72-hour cumulative incidence for IM medication as 17% (Custody), 6% (Report), and 5% (None), and physical restraints as 8%, 4%, and 3%. Results Text / Table 2: Table 2 lists the actual raw percentages for IM Medication used as 7.3% (Custody), 4.0% (Report), and 2.8% (None), and Physical Restraints as 4.8%, 2.5%, and 1.4%.  Clarification Needed: The cumulative incidence derived from a survival analysis/Cox curve accounts for censoring and time-to-event probabilities over 72 hours, which explains why they differ from raw proportions. However, the text in the Results section (paragraphs 3 and 5) states: "Finally at 72 hours, the number of encounters increased to 146 (5%), 18 (6%), and 31 (17%) respectively." This reads as if 31 is 17% of 436, which is mathematically incorrect (31 / 436 = 7.1\%).  Action Required: Explicitly clarify in the text when you are reporting the raw sample proportion versus the model-estimated cumulative incidence probability at the 72-hour mark. | We appreciate the reviewer’s comment to clarify this confusion. In response, we clarified that Table 2 describes raw percentages only whereas in the manuscript, when we describe the results from our models, we are presenting cumulative incidences. | We note that Table 2 features raw percentages for the “IM Medication Used” and “Physical Restraints Used” rows and explicitly state when we are presenting cumulative incidences **on lines 187 – 193** and **on lines 209 – 215**. |
| 3. Elaborate on the Proportional Hazards Assumption  In the Methods section, the authors state: "The proportional hazards assumption was evaluated using Schoenfeld residuals."  Action Required: State the results of this evaluation. Did any covariates violate the assumption? If so, how was it managed (e.g., time-varying covariates)? Given that length of stay varies widely, ensuring the hazard ratio remains constant over the 72-hour window is vital for the integrity of the Cox models. | Thank you for bringing this to our attention. We re-ran this analysis to confirm the proportional hazards assumption was not violated by any of the covariates in both of our Cox models and explicitly state this result in the manuscript. | We state the results of the testing the proportional hazard assumption in the methods section on **lines 147 – 148.** |
| Minor Revisions | | |
| 1. Justification of the 30-Minute Restraint Cutoff  Critique: The authors categorize restraint timing into <30 minutes and >30 minutes based on clinical intuition regarding pre-hospital vs. ED-driven factors.  Action Required: Provide a brief clinical or literature-based justification for why exactly 30 minutes was chosen as the threshold for an "ED-driven environment" vs. an "arrival transition" effect. | We appreciate the reviewer’s request for additional justification of the 30-minute threshold. The cutoff was selected a priori based on clinical and operational considerations.  Clinically, restraint events occurring shortly after ED arrival are often influenced by pre-hospital circumstances, transportation-related factors, or the transition into the ED environment, leaving limited opportunity for ED-based interventions to affect outcomes. We considered the first 30 minutes after arrival to represent a transition period during which restraint use is less likely to reflect ED-specific processes. In addition, examination of the distribution of time from arrival to restraint demonstrated a natural clustering of early restraint events within this time frame.  We acknowledge that any temporal cutoff is somewhat arbitrary and that documentation imprecision may also influence the exact timing of restraints events. However, we selected 30 minutes as a clinically reasonable threshold that balanced these considerations while allowing distinction between early restraint use likely related to pre-arrival factors and later restraint use more likely to influenced by ED care processes. | We added our rationale for this cutoff in the methods section on **lines 122 – 128.** |
| Conclusion of Review  This study provides incredibly actionable insight into pediatric healthcare equity. If the authors can cleanly reconcile their statistical reporting definitions and directly address the conspicuous intersection of race and child welfare status in their data, this manuscript will make an excellent addition to PLOS ONE. | Thank you for your review conclusion of our study. We believe that incorporating your major and minor revisions greatly strengthened our study for resubmission. | No changes. |
