# Supplementary material for: Children in child protective services custody experience higher risk of intramuscular medication and physical restraints in a pediatric emergency department
Source: PLoS One. 2026 Aug 12;21(8):e0355397. doi: 10.1371/journal.pone.0355397 (PMC13465842; doi:10.1371/journal.pone.0355397)
Supplement: Peer review history

# Peer review: Decision Letter 0

**DOI:** 10.1371/journal.pone.0355397.r001

**Authors:** Souparno Mitra

2 Jun 2026

Dear Dr. Faustino,

Thank you for submitting your manuscript to PLOS ONE. After careful consideration, we feel that it has merit but does not fully meet PLOS ONE’s publication criteria as it currently stands. Therefore, we invite you to submit a revised version of the manuscript that addresses the points raised during the review process.

==============================

**ACADEMIC EDITOR:** **Please address reviewers comments and resubmit for further consideration for publication.**

==============================

Please submit your revised manuscript by Jul 17 2026 11:59PM. If you will need more time than this to complete your revisions, please reply to this message or contact the journal office at plosone@plos.org. When you're ready to submit your revision, log on to https://www.editorialmanager.com/pone/ and select the 'Submissions Needing Revision' folder to locate your manuscript file.

- A letter that responds to each point raised by the academic editor and reviewer(s). You should upload this letter as a separate file labeled 'Response to Reviewers'.
- A marked-up copy of your manuscript that highlights changes made to the original version. You should upload this as a separate file labeled 'Revised Manuscript with Track Changes'.
- An unmarked version of your revised paper without tracked changes. You should upload this as a separate file labeled 'Manuscript'.

If you would like to make changes to your financial disclosure, please include your updated statement in your cover letter. Guidelines for resubmitting your figure files are available below the reviewer comments at the end of this letter.

If applicable, we recommend that you deposit your laboratory protocols in protocols.io to enhance the reproducibility of your results. Protocols.io assigns your protocol its own identifier (DOI) so that it can be cited independently in the future. For instructions see: https://journals.plos.org/plosone/s/submission-guidelines#loc-laboratory-protocols. Additionally, PLOS ONE offers an option for publishing peer-reviewed Lab Protocol articles, which describe protocols hosted on protocols.io. Read more information on sharing protocols at https://plos.org/protocols?utm_medium=editorial-email&utm_source=authorletters&utm_campaign=protocols.

As the corresponding author, your ORCID iD is verified in the submission system and will appear in the published article. PLOS supports the use of ORCID, and we encourage all coauthors to register for an ORCID iD and use it as well. Please encourage your coauthors to verify their ORCID iD within the submission system before final acceptance, as unverified ORCID iDs will not appear in the published article. *Only*  the individual author can complete the verification step; PLOS staff *cannot*  verify ORCID iDs on behalf of authors.

We look forward to receiving your revised manuscript.

Kind regards,

Souparno Mitra, M.D.

Academic Editor

PLOS One

Journal Requirements:

When submitting your revision, we need you to address these additional requirements.

1. Please ensure that your manuscript meets PLOS ONE's style requirements, including those for file naming. The PLOS ONE style templates can be found at

https://journals.plos.org/plosone/s/file?id=wjVg/PLOSOne_formatting_sample_main_body.pdf and https://journals.plos.org/plosone/s/file?id=ba62/PLOSOne_formatting_sample_title_authors_affiliations.pdf

2. We note that you have indicated that there are restrictions to data sharing for this study. For studies involving human research participant data or other sensitive data, we encourage authors to share de-identified or anonymized data. However, when data cannot be publicly shared for ethical reasons, we allow authors to make their data sets available upon request. For information on unacceptable data access restrictions, please see http://journals.plos.org/plosone/s/data-availability#loc-unacceptable-data-access-restrictions.

Before we proceed with your manuscript, please address the following prompts:

a) If there are ethical or legal restrictions on sharing a de-identified data set, please explain them in detail (e.g., data contain potentially identifying or sensitive patient information, data are owned by a third-party organization, etc.) and who has imposed them (e.g., a Research Ethics Committee or Institutional Review Board, etc.). Please also provide contact information for a data access committee, ethics committee, or other institutional body to which data requests may be sent.

b) If there are no restrictions, please upload the minimal anonymized data set necessary to replicate your study findings to a stable, public repository and provide us with the relevant URLs, DOIs, or accession numbers. Please see http://www.bmj.com/content/340/bmj.c181.long for guidelines on how to de-identify and prepare clinical data for publication. For a list of recommended repositories, please see https://journals.plos.org/plosone/s/recommended-repositories. You also have the option of uploading the data as Supporting Information files, but we would recommend depositing data directly to a data repository if possible.

Please update your Data Availability statement in the submission form accordingly.

3. In the online submission form, you indicated that data set will be available upon request after complete deidentification.

All PLOS journals now require all data underlying the findings described in their manuscript to be freely available to other researchers, either 1. In a public repository, 2. Within the manuscript itself, or 3. Uploaded as supplementary information.

This policy applies to all data except where public deposition would breach compliance with the protocol approved by your research ethics board. If your data cannot be made publicly available for ethical or legal reasons (e.g., public availability would compromise patient privacy), please explain your reasons on resubmission and your exemption request will be escalated for approval.

4. Please amend your authorship list in your manuscript file to include author Isaac Faustino.

5. Please amend the manuscript submission data (via Edit Submission) to include author Isaac V. Faustino.

6. If the reviewer comments include a recommendation to cite specific previously published works, please review and evaluate these publications to determine whether they are relevant and should be cited. There is no requirement to cite these works unless the editor has indicated otherwise.

[Note: HTML markup is below. Please do not edit.]

Reviewers' comments:

Reviewer's Responses to Questions

**Comments to the Author**

1. Is the manuscript technically sound, and do the data support the conclusions?

Reviewer #1: Yes

Reviewer #2: Yes

**********

2. Has the statistical analysis been performed appropriately and rigorously?

Reviewer #1: Yes

Reviewer #2: Yes

**********

3. Have the authors made all data underlying the findings in their manuscript fully available?

The PLOS Data policy

Reviewer #1: No

Reviewer #2: No

**********

4. Is the manuscript presented in an intelligible fashion and written in standard English?

Reviewer #1: Yes

Reviewer #2: Yes

**********

Reviewer #1: The authors conducted a single center retrospective cohort study using electronic health records from pediatric ED encounters, showing that children in CPS custody had higher risk of receiving IM medication and physical restraint during BH encounters.

I appreciate the focus on the important topic of children under CPS care and the unique strength of the time-to-event analysis.

My primary concern with the findings is confounding by acuity. In other words, are children in CPS or children that receive a CPS consult more acutely or chronically ill and therefore restrictive interventions were clinically warranted/justified in such cases? I understand the authors’ position that this paper is an initial step and not, in theory, pointing to causality, but the discussion focuses on the hypothesis that CPS care places children at risk for restrictive interventions (which might be true) but spends less time on the idea that children placed within CPS care are already more acutely and chronically ill in the first place. It would be interesting to see if those who have a CPS consult or are in CPS custody have a history of restraint or IM sedation in prior visits. If not, this would strengthen the authors contention.

I have also added minor comments below which I hope strengthen the paper.

1. In table 1 can the authors explain the inclusion of “hyperventilating?” Seems like there would be a broad overlap with acute medical presentations.

2. Can the authors give one line as to why you chose to group CPS custody + new report into the custody group vs. creating a fourth group? At minimum, could authors report how many of these there were?

3. I appreciate the authors using manual review of charts to determine timing of physical restraint use. Is there precedent for this? Curious to know how accurate/validated this methodology is?

Reviewer #2: Overall, this is a well-written, clinically crucial, and methodologically sound retrospective cohort study. It addresses a major gap in pediatric emergency medicine and child welfare literature by breaking down "CPS involvement" into nuanced categories (custody vs. new report) and utilizing time-to-event analysis.

The review below is organized into major and minor revisions to help the authors improve the manuscript before publication.

Reviewer Scientific Evaluation Report

Recommendation: Major Revision

General Assessment

The manuscript tackles an incredibly vital topic—the intersection of child welfare status and restrictive clinical interventions (physical restraints and intramuscular chemical restraints) in pediatric emergency departments. The use of Cox proportional hazards models with patient-specific frailty terms to account for repeat visits is a robust and appropriate statistical choice.

However, there is a striking demographic signal in Table 2 regarding Race and Ethnicity that requires immediate attention. The authors adjust for race/ethnicity in their regression models as a confounder, but they completely omit any discussion of how racial disparities overlap with CPS custody status and restraint use in their Discussion section. This must be addressed to ensure the study provides a complete, systems-level perspective.

Major Revisions

1. Address Intersectionality and Racial Disparities in the Discussion

In Table 2, the demographic breakdown of the cohorts reveals a severe racial imbalance across the levels of CPS involvement:

White non-Hispanic: Dropping from 44.5% (No CPS) to 25.0% (CPS Custody).

Black non-Hispanic: Surging from 21.6% (No CPS) to 42.0% (CPS Custody).

We know from existing literature (including the authors' cited reference, Nash et al., 2021) that Black youth face a disproportionately higher risk of being physically restrained in emergency settings. By adjusting for race in the Cox model, the authors show that CPS custody carries an independent risk (aHR = 2.72 for restraints), but they fail to discuss how systemic racism in child welfare pipelines concentrates highly vulnerable Black youth into this highest-risk "CPS Custody" cohort.

Action Required: Add a dedicated subsection or paragraph in the Discussion exploring this intersection. The paper cannot fully evaluate "systems-level interventions" without acknowledging how structural racism impacts both child welfare entry and clinical management in behavioral crises.

2. Discrepancy Between Abstract and Results Text

There is a confusing numerical discrepancy between the cumulative incidence rates reported in the Abstract versus the Results section text:

Abstract: Reports 72-hour cumulative incidence for IM medication as 17% (Custody), 6% (Report), and 5% (None), and physical restraints as 8%, 4%, and 3%.

Results Text / Table 2: Table 2 lists the actual raw percentages for IM Medication used as 7.3% (Custody), 4.0% (Report), and 2.8% (None), and Physical Restraints as 4.8%, 2.5%, and 1.4%.

Clarification Needed: The cumulative incidence derived from a survival analysis/Cox curve accounts for censoring and time-to-event probabilities over 72 hours, which explains why they differ from raw proportions. However, the text in the Results section (paragraphs 3 and 5) states: "Finally at 72 hours, the number of encounters increased to 146 (5%), 18 (6%), and 31 (17%) respectively." This reads as if 31 is 17% of 436, which is mathematically incorrect (31 / 436 = 7.1\%).

Action Required: Explicitly clarify in the text when you are reporting the raw sample proportion versus the model-estimated cumulative incidence probability at the 72-hour mark.

3. Elaborate on the Proportional Hazards Assumption

In the Methods section, the authors state: "The proportional hazards assumption was evaluated using Schoenfeld residuals." * Action Required: State the results of this evaluation. Did any covariates violate the assumption? If so, how was it managed (e.g., time-varying covariates)? Given that length of stay varies widely, ensuring the hazard ratio remains constant over the 72-hour window is vital for the integrity of the Cox models.

Minor Revisions

1. Justification of the 30-Minute Restraint Cutoff

Critique: The authors categorize restraint timing into <30 minutes and >30 minutes based on clinical intuition regarding pre-hospital vs. ED-driven factors.

Action Required: Provide a brief clinical or literature-based justification for why exactly 30 minutes was chosen as the threshold for an "ED-driven environment" vs. an "arrival transition" effect.

Conclusion of Review

This study provides incredibly actionable insight into pediatric healthcare equity. If the authors can cleanly reconcile their statistical reporting definitions and directly address the conspicuous intersection of race and child welfare status in their data, this manuscript will make an excellent addition to PLOS ONE.

**********

what does this mean?). If published, this will include your full peer review and any attached files.

If you choose “no”, your identity will remain anonymous but your review may still be made public.

**Do you want your identity to be public for this peer review?**  For information about this choice, including consent withdrawal, please see our Privacy Policy

Reviewer #1: No

Reviewer #2: **Yes:**  Dr Rimah Melati Ab Ghani

**********

[NOTE: If reviewer comments were submitted as an attachment file, they will be attached to this email and accessible via the submission site. Please log into your account, locate the manuscript record, and check for the action link "View Attachments". If this link does not appear, there are no attachment files.]

To ensure your figures meet our technical requirements, please review our figure guidelines: https://journals.plos.org/plosone/s/figures

You may also use PLOS’s free figure tool, NAAS, to help you prepare publication quality figures: https://journals.plos.org/plosone/s/figures#loc-tools-for-figure-preparation.

NAAS will assess whether your figures meet our technical requirements by comparing each figure against our figure specifications.

# Author comment: Author response to Decision Letter 1

**DOI:** 10.1371/journal.pone.0355397.r002

1 Jul 2026

Reviewer #1:

"My primary concern with the findings is confounding by acuity. In other words, are children in CPS or children that receive a CPS consult more acutely or chronically ill and therefore restrictive interventions were clinically warranted/justified in such cases?

I understand the authors’ position that this paper is an initial step and not, in theory, pointing to causality, but the discussion focuses on the hypothesis that CPS care places children at risk for restrictive interventions (which might be true) but spends less time on the idea that children placed within CPS care are already more acutely and chronically ill in the first place.

It would be interesting to see if those who have a CPS consult or are in CPS custody have a history of restraint or IM sedation in prior visits. If not, this would strengthen the authors contention."

We appreciate the reviewer’s thoughtful comment. We agree that children in CPS custody or children with new CPS referrals may have more frequent and severe BH dysregulation requiring additional restrictive interventions.

Per the reviewer’s recommendation, we examined patients who had at least 2 encounters in our health system and analyzed whether the CPS grouping of a patient’s most recent encounter was associated with having a prior IM medication or physical restraint event. We found that 18 patients (23.4% of 77 unique patients in CPS Custody), 13 patients (12.4% of 105 unique patients with only a new CPS report), and 45 patients (5.2% of 867 unique patients with no CPS involvement) had a prior history of IM medication or physical restraint. A chi-square test revealed that CPS grouping and prior history of IM medication or physical restraint were associated (p<0.001).

This supplemental analysis suggests that youth in CPS custody and those with new CPS referrals were significantly more likely to have a prior history of IM medication administration or physical restraint than youth without CPS Involvement. This finding supports the possibility that these populations enter emergency care settings with greater baseline behavioral health complexity and vulnerability. However, such differences may themselves reflect the cumulative effects of adverse childhood experiences, maltreatment exposure, placement instability, and fragmented access to behavioral health services that are disproportionately experienced by CPS-involved youth. Therefore, we believe that greater clinical acuity and structural vulnerability should be viewed as complementary rather than competing explanations for the observed associations.

1. "In table 1 can the authors explain the inclusion of “hyperventilating?” Seems like there would be a broad overlap with acute medical presentations."

Hyperventilating was used as a chief complaint to capture instances of acute anxiety. We removed 25 encounters from the analysis dataset that listed “hyperventilating” as the primary chief complaint but did not have an accompanying acute anxiety chief complaint.

2. "Can the authors give one line as to why you chose to group CPS custody + new report into the custody group vs. creating a fourth group? At minimum, could authors report how many of these there were?"

We appreciate the reviewer’s suggestion. We categorized encounters involving children already in CPS custody and a new CPS report within the CPS custody group because CPS custody represents the child’s existing legal status and care environment at the time of the ED encounter. Although a new report may be filed, these children remain in CPS custody and continue to share key characteristics relevant to our study question, including the same guardian/legal decision-maker structure, placement-related challenges, fractured behavioral healthcare, and child welfare oversight. Therefore, we considered CPS custody to be the dominant exposure category.

Of the 436 encounters involving patients in CPS custody, 185 (42.4%) also had a new CPS report filed during the encounter.

3. "I appreciate the authors using manual review of charts to determine timing of physical restraint use. Is there precedent for this? Curious to know how accurate/validated this methodology is?"

There is precedence for using manual chart review to identify and characterize restraint events, although this approach is not uniformly described or validated across studies. In a recent systematic review of restrictive care practices, a subset of studies used manual review of medical records to extract restraint-related data, including timing and duration of restraint episodes (Belayneh et al. 2024). However, the review also highlighted substantial variability in reporting and a lack of formal validation of measurement approaches across studies.

In our study, we used manual chart review to determine the timing of physical restraint use because structured elements alone did not consistently capture this information. For each encounter, in which physical restraint use was documented, a study team member (IVF) reviewed clinician and nursing documentation to verify whether restraints were used and to identify the timing of the restraint event relative to the emergency department encounter. In cases where the timing was unclear or not explicitly documented, a second reviewer (MJR) independently reviewed the same documentation and discrepancies were resolved by consensus.

Reviewer #2:

"1. Address Intersectionality and Racial Disparities in the Discussion

In Table 2, the demographic breakdown of the cohorts reveals a severe racial imbalance across the levels of CPS involvement:

White non-Hispanic: Dropping from 44.5% (No CPS) to 25.0% (CPS Custody).

Black non-Hispanic: Surging from 21.6% (No CPS) to 42.0% (CPS Custody).

We know from existing literature (including the authors' cited reference, Nash et al., 2021) that Black youth face a disproportionately higher risk of being physically restrained in emergency settings. By adjusting for race in the Cox model, the authors show that CPS custody carries an independent risk (aHR = 2.72 for restraints), but they fail to discuss how systemic racism in child welfare pipelines concentrates highly vulnerable Black youth into this highest-risk "CPS Custody" cohort.

Action Required: Add a dedicated subsection or paragraph in the Discussion exploring this intersection. The paper cannot fully evaluate "systems-level interventions" without acknowledging how structural racism impacts both child welfare entry and clinical management in behavioral crises."

We appreciate the reviewer’s suggestion to incorporate information exploring how racial inequities leads to Black youth disproportionately being concentrated within CPS custody.

We revisited the literature to write additional information about this phenomenon and how it connects to our study’s findings. We have included additional information describing how Black youth have disproportionately higher rates of CPS involvement and how this is associated with higher rates of IM medication and physical restraint use within the pediatric ED environment in the discussion section on lines 274 - 280.

"2. Discrepancy Between Abstract and Results Text

There is a confusing numerical discrepancy between the cumulative incidence rates reported in the Abstract versus the Results section text:

Abstract: Reports 72-hour cumulative incidence for IM medication as 17% (Custody), 6% (Report), and 5% (None), and physical restraints as 8%, 4%, and 3%.

Results Text / Table 2: Table 2 lists the actual raw percentages for IM Medication used as 7.3% (Custody), 4.0% (Report), and 2.8% (None), and Physical Restraints as 4.8%, 2.5%, and 1.4%.

Clarification Needed: The cumulative incidence derived from a survival analysis/Cox curve accounts for censoring and time-to-event probabilities over 72 hours, which explains why they differ from raw proportions. However, the text in the Results section (paragraphs 3 and 5) states: "Finally at 72 hours, the number of encounters increased to 146 (5%), 18 (6%), and 31 (17%) respectively." This reads as if 31 is 17% of 436, which is mathematically incorrect (31 / 436 = 7.1\%).

Action Required: Explicitly clarify in the text when you are reporting the raw sample proportion versus the model-estimated cumulative incidence probability at the 72-hour mark."

We appreciate the reviewer’s comment to clarify this confusion. In response, we clarified that Table 2 describes raw percentages only whereas in the manuscript, when we describe the results from our models, we are presenting cumulative incidences. We note that Table 2 features raw percentages for the “IM Medication Used” and “Physical Restraints Used” rows and explicitly state when we are presenting cumulative incidences on lines 187 – 193 and on lines 209 – 215.

"3. Elaborate on the Proportional Hazards Assumption

In the Methods section, the authors state: "The proportional hazards assumption was evaluated using Schoenfeld residuals."

Action Required: State the results of this evaluation. Did any covariates violate the assumption? If so, how was it managed (e.g., time-varying covariates)? Given that length of stay varies widely, ensuring the hazard ratio remains constant over the 72-hour window is vital for the integrity of the Cox models."

Thank you for bringing this to our attention. We re-ran this analysis to confirm the proportional hazards assumption was not violated by any of the covariates in both of our Cox models and explicitly state this result in the manuscript. We state the results of the testing the proportional hazard assumption in the methods section on lines 147 – 148.

"1. Justification of the 30-Minute Restraint Cutoff

Critique: The authors categorize restraint timing into <30 minutes and >30 minutes based on clinical intuition regarding pre-hospital vs. ED-driven factors.

Action Required: Provide a brief clinical or literature-based justification for why exactly 30 minutes was chosen as the threshold for an "ED-driven environment" vs. an "arrival transition" effect."

We appreciate the reviewer’s request for additional justification of the 30-minute threshold. The cutoff was selected a priori based on clinical and operational considerations.

Clinically, restraint events occurring shortly after ED arrival are often influenced by pre-hospital circumstances, transportation-related factors, or the transition into the ED environment, leaving limited opportunity for ED-based interventions to affect outcomes. We considered the first 30 minutes after arrival to represent a transition period during which restraint use is less likely to reflect ED-specific processes. In addition, examination of the distribution of time from arrival to restraint demonstrated a natural clustering of early restraint events within this time frame.

We acknowledge that any temporal cutoff is somewhat arbitrary and that documentation imprecision may also influence the exact timing of restraints events. However, we selected 30 minutes as a clinically reasonable threshold that balanced these considerations while allowing distinction between early restraint use likely related to pre-arrival factors and later restraint use more likely to influenced by ED care processes. We added our rationale for this cutoff in the methods section on lines 122 – 128.

Attachment Submitted filename: Response to Reviewers.docx

# Peer review: Decision Letter 1

**DOI:** 10.1371/journal.pone.0355397.r003

**Authors:** Souparno Mitra

21 Jul 2026

Children in Child Protective Services Custody Experience Higher Risk of Intramuscular Medication and Physical Restraints in a Pediatric Emergency Department

PONE-D-26-14643R1

Dear Dr. Faustino,

We’re pleased to inform you that your manuscript has been judged scientifically suitable for publication and will be formally accepted for publication once it meets all outstanding technical requirements.

Within one week, you’ll receive an e-mail detailing the required amendments. When these have been addressed, you’ll receive a formal acceptance letter and your manuscript will be scheduled for publication.

An invoice will be generated when your article is formally accepted. Please note, if your institution has a publishing partnership with PLOS and your article meets the relevant criteria, all or part of your publication costs will be covered. Please make sure your user information is up-to-date by logging into Editorial Manager at Editorial Manager® and clicking the ‘Update My Information' link at the top of the page. For questions related to billing, please contact billing support.

If your institution or institutions have a press office, please notify them about your upcoming paper to help maximize its impact. If they’ll be preparing press materials, please inform our press team as soon as possible -- no later than 48 hours after receiving the formal acceptance. Your manuscript will remain under strict press embargo until 2 pm Eastern Time on the date of publication. For more information, please contact onepress@plos.org.

Kind regards,

Souparno Mitra, M.D.

Academic Editor

PLOS One

Additional Editor Comments (optional):

Reviewers' comments:

Reviewer's Responses to Questions

**Comments to the Author**

Reviewer #1: All comments have been addressed

Reviewer #2: All comments have been addressed

**********

2. Is the manuscript technically sound, and do the data support the conclusions?

Reviewer #1: Yes

Reviewer #2: Yes

**********

3. Has the statistical analysis been performed appropriately and rigorously?

Reviewer #1: Yes

Reviewer #2: Yes

**********

4. Have the authors made all data underlying the findings in their manuscript fully available?

The PLOS Data policy

Reviewer #1: Yes

Reviewer #2: Yes

**********

5. Is the manuscript presented in an intelligible fashion and written in standard English?

Reviewer #1: Yes

Reviewer #2: Yes

**********

Reviewer #1: Thank you for the opportunity to review this revision. The authors have addressed my concerns and I have no further comments

Reviewer #2: Manuscript ID: PONE-D-26-14643R1

Title: Children in Child Protective Services Custody Experience Higher Risk of Intramuscular Medication and Physical Restraints in a Pediatric Emergency Department

A. Recommendation: Accept

B. General Assessment & Overview

This revised manuscript addresses a highly critical and historically under-investigated topic within pediatric emergency medicine and child welfare: the intersection of Child Protective Services (CPS) custody status and the deployment of highly restrictive clinical interventions (intramuscular chemical sedatives and physical restraints). The distinct separation between children with an acute new report filed versus those already in CPS custody represents a valuable nuance that highlights systems-level vulnerabilities rather than treating all child welfare interactions as a monolith.

The authors’ utilization of a retrospective time-to-event analysis (Cox proportional hazards regression with patient-specific frailty terms) is methodologically robust for handling repeat emergency department (ED) encounters. In this revised version (R1), the authors have meticulously and satisfactorily addressed the critiques raised during the initial round of peer review. The clarity, structural integrity, and clinical discussion of the paper are significantly improved.

C. Evaluation of Responses to Reviewer Critiques

1. Structural Racism and Intersectionality

1.1 Author Action: The authors incorporated a dedicated discussion track (lines 274–280) evaluating the severe demographic imbalances visible in Table 2, where Black non-Hispanic youth surge from 21.6% of the baseline cohort to 42.0% of the CPS Custody cohort.

1.2 Reviewer Appraisal: Acknowledging the child welfare pipeline and systemic racism's contribution to placing highly vulnerable minority youth into cohorts facing independent risks of restraint (aHR = 2.712) significantly elevates the real-world clinical utility and equity perspective of this paper.

2. Statistical Clarity

2.1 Author Action: The numerical text in the Results section was successfully revised to prevent readers from confusing raw sample proportions (e.g., 7.3% raw IM medication use in CPS custody) with the model-estimated cumulative incidence probabilities calculated across the full 72-hour survival curve window (17% at 72 hours). They also explicitly stated the validation metrics of the Schoenfeld residuals confirming the proportional hazards assumption (lines 147–148).

2.2 Reviewer Appraisal: The data presentation is now mathematically precise and adheres strictly to epidemiological standards for reporting survival metrics.

D. Final Conclusion & Recommendation

The authors have demonstrated extreme diligence in executing necessary data restructuring, adding analytical clarity, and contextually deepening their narrative regarding child health equity. The paper is highly actionable, methodologically sound, and matches the rigorous indexing standards of PLOS ONE.

I recommend this manuscript for publication.

**********

what does this mean?). If published, this will include your full peer review and any attached files.

If you choose “no”, your identity will remain anonymous but your review may still be made public.

**Do you want your identity to be public for this peer review?**  For information about this choice, including consent withdrawal, please see our Privacy Policy

Reviewer #1: No

Reviewer #2: **Yes:**  Dr Rimah Melati Ab Ghani

**********

# Editor report: Acceptance letter

**DOI:** 10.1371/journal.pone.0355397.r004

**Authors:** Souparno Mitra

PONE-D-26-14643R1

PLOS One

Dear Dr. Faustino,

I'm pleased to inform you that your manuscript has been deemed suitable for publication in PLOS One. Congratulations! Your manuscript is now being handed over to our production team.

At this stage, our production department will prepare your paper for publication. This includes ensuring the following:

* All references, tables, and figures are properly cited

* All relevant supporting information is included in the manuscript submission,

* There are no issues that prevent the paper from being properly typeset

You will receive further instructions from the production team, including instructions on how to review your proof when it is ready. Please keep in mind that we are working through a large volume of accepted articles, so please give us a few days to review your paper and let you know the next and final steps.

Lastly, if your institution or institutions have a press office, please let them know about your upcoming paper now to help maximize its impact. If they'll be preparing press materials, please inform our press team within the next 48 hours. Your manuscript will remain under strict press embargo until 2 pm Eastern Time on the date of publication. For more information, please contact onepress@plos.org.

You will receive an invoice from PLOS for your publication fee after your manuscript has reached the completed accept phase. If you receive an email requesting payment before acceptance or for any other service, this may be a phishing scheme. Learn how to identify phishing emails and protect your accounts at https://explore.plos.org/phishing.

If we can help with anything else, please email us at customercare@plos.org.

Thank you for submitting your work to PLOS ONE and supporting open access.

Kind regards,

PLOS ONE Editorial Office Staff

on behalf of

Dr. Souparno Mitra

Academic Editor

PLOS One
